# Absent Treatments

**Published:** 1899-09

**Authors:** 


### "Absent Treatments."

The *Massachusetts Medical Journal* is responsible for the following: A man of the Christian Science faith fractured his femur. Under Christian Science it united, of course, but there was considerable shortening. Some months later a lady called at his place of business to sell him a book. In the course of conversation he related his experience, bemoaning the fact that the one-timed injured limb was so short that he walked with much difficulty. She expressed much sympathy, but assured him that just as Christian Science had mended the bone, just so could it lengthen it. She informed him that she understood and could give him one treatment then, and after that give him what was called "absent treatment." This she did and departed. A fortnight later the man believed his leg was really a little longer. After another week later, still longer, and soon after it was long as the other, and later still it was longer than the uninjured limb. At last accounts the leg was getting too long; the "absent treatment" was still going on, and the whereabouts of the woman could not be ascertained.—*American Medical Compend.*

["Pulling his leg?"]

